# Primary neuroendocrine breast carcinomas are associated with poor local control despite favourable biological profile: a retrospective clinical study

**DOI:** 10.1186/s12885-017-3056-4

**Published:** 2017-01-24

**Authors:** Nelli Roininen, Sari Takala, Kirsi-Maria Haapasaari, Arja Jukkola-Vuorinen, Johanna Mattson, Päivi Heikkilä, Peeter Karihtala

**Affiliations:** 1 Department of Oncology and Radiotherapy, Medical Research Center Oulu, Oulu University Hospital and University of Oulu, P.O. Box 22, FIN-90029 Oulu, Finland; 2 Department of Pathology, Medical Research Center Oulu, Oulu University Hospital and University of Oulu, Oulu, Finland; 3 Helsinki University Hospital Comprehensive Cancer Center, Helsinki, Finland; 4 Department of Pathology, University of Helsinki and Helsinki University Hospital, Helsinki, Finland

**Keywords:** Breast cancer, Incidence, Neuroendocrine carcinoma, Prognosis, World Health Organization

## Abstract

**Background:**

Breast carcinomas with neuroendocrine features (NEBC) are a very rare entity of mammary neoplasms, WHO classification of which has recently been revised. There are very limited data available about the clinical behaviour and treatment options of NEBC.

**Methods:**

We collected retrospectively patients with NEBC from Oulu and Helsinki University Hospitals in 2007–2015. There were 43 NEBC cases during the period.

**Results:**

The incidence of NEBC from all breast cancers varied from 0.1% in Helsinki to 1.3% in Oulu. The mean tumor size was 2.2 cm and 23 patients (55.8%) had no lymph node metastases when diagnosed. In total, 4 patients (9.3%) had distant metastases at the time of diagnosis. High estrogen receptor (ER) expression was observed in 41 (97.7%) patients. When non-metastatic NEBC were compared to a prospective set of ductal carcinomas (n = 506), NEBC were more often HER2 negative (*p =* 0.046), ER positive (*p =* 0.0062) and the NEBC patients were older (*p* < 0.0005) than patients with ductal carcinomas. Plasma chromogranin A correlated only to higher age at diagnosis (*p =* 0.0028). Relapse-free survival (*p =* 0.0013), disease-free survival (*p =* 0.024) and overall survival (*p =* 0.0028) favoured ductal carcinomas compared to NEBC, while no difference was observed in distant disease-free survival or in breast cancer-specific survival.

**Conclusions:**

There is remarkable variation in the incidence of NEBC in Finland, which is likely to be explained by differences in the use of neuroendocrine marker immunostainings. Poor local control and worse overall survival may be linked to the more aggressive biology of the disease, despite its association with apparently indolent prognostic factors.

## Background

Breast carcinomas with neuroendocrine features (NEBC) are usually estimated to represent <1% of all breast cancers [1, 2]. Their actual incidence is in fact difficult to assess since neuroendocrine markers (NE) are not routinely used in breast cancer diagnostics and NEBC are challenging to recognize clinically or with basic immunohistochemistry. The World Health Organization (WHO) definition for breast cancer with neuroendocrine features has been revised lately [1, 3]. According to the definition of 2012, there is no specific threshold for the positivity of neuroendocrine marker expression, namely synaptophysin or chromogranin but either expression is required for the diagnosis. According to WHO, chromogranin and synaptophysin expression may significantly vary, depending e.g. on the grade. The latest WHO diagnostic criteria for NEBC also emphasize the reliable exclusion of the possibility of metastatic neuroendocrine/small-cell carcinoma before a definite diagnosis since ≥97% of all neuroendocrine carcinomas derive from lungs or gastrointestinal tract [4]. The presence of ductal carcinoma in situ (DCIS) is supportive of origin in the breast [1]. Clinically and macroscopically NEBCs are indistinguishable from other tumour types [1].

According to the most common theory, NEBC are thought to originate from neoplastic epithelial cells during the carcinogenesis [5]. There might still be prognostic and predictive differences between the NEBC and ductal carcinomas – some papers using older criteria for NEBC have suggested different biological profile and maybe also poorer prognosis of the patients with NEBC [6, 7]. Data from small series suggest that NEBC comprise a discrete molecular cluster [8]. When a large set of mucinous breast carcinomas were compared with NEBCs, hypercellular mucinous B type tumours had a very close profile both in hierarchical clustering analysis and in transcriptomic analysis. In contrast, grade- and molecular subtype-matched invasive ductal carcinomas were transcriptionally distinct from NEBCs [9]. Some NEBC also harbour potentially actionable oncogene mutations such as PI3KCA, FGRF and RAS mutations [8].

We collected retrospectively NEBC cases and their clinicopathological data from two Finnish University Hospitals from the past 8.5 years. Special emphasis was given to follow the current WHO criteria for NEBC, in particular the exclusion of primary tumor from extramammary sites and adequate NE marker expression. Our primary aim was to compare the prognostic factor profile of NEBC to the prospective contemporary set of ductal carcinomas. Collection of NEBC cases from two large hospitals also allowed us to assess the possible differences in the incidence of NEBC within the country.

## Methods

We collected retrospectively patients diagnosed with NEBC from Oulu and Helsinki University Hospitals. The electronic database of the Departments of Pathology was searched from January 2007 (database launch) to July 2015 using the same criteria in both hospitals. To exclude extramammary origin of carcinoma, the cases without either 1) both abdominal and thoracic imaging at the time of diagnosis or 2) histological identification of ductal in situ carcinoma (DCIS) component were excluded [1]. Patient data was collected from the medical records of Oulu and Helsinki University Hospitals. The comparison group consisted of 506 local or locally advanced ductal invasive carcinomas from the prospective cohort diagnosed and treated in Oulu University Hospital in 2003–2011.

The histopathology of ductal carcinomas was evaluated after current WHO classification and patients were classed after their TNM classification [1]. Estrogen receptor (ER), progesterone receptor (PR) and Ki-67 expressions were studied by immunohistochemistry as described previously [10]. Grading of NEBC was omitted as suggested by WHO criteria [1]. HER2 expression was studied by immunohistochemistry and when HER2-positive result appeared, gene amplification status was determined using chromogenic in situ hybridization. Cancers with six or more gene copies were considered *HER2* positive [11]. Plasma chromogranin A (CgA) was measured radioimmunologically at the time of diagnosis in 15 NEBC patients. Synaptophysin and chromogranin immunohistochemical expression was considered positive when there was expression of either marker in >50% of tumor cells.

## Statistical analyses

Statistical analysis was performed using IBM SPSS Statistics software, v. 22.0.0.0 (IBM Corporation, Armonk, NY, USA). The significance of associations was defined by using two-sided Pearson's Chi-square test or Fisher's exact test if available. Mann Whitney *U* test was used when assessing the continuous variables (age or plasma CgA). Spearman’s test with correlation coefficient was applied when correlating plasma CgA to age. Kaplan–Meier curves with the log-rank test were applied in survival analysis. Disease-free (DFS), relapse-free (RFS), distant disease-free (DDFS), breast cancer-specific (BCSS) and overall (OS) survival were calculated from the time of diagnosis to disease recurrence at any site (DFS), in the ipsilateral breast, scar, or axilla (RFS), at distant sites (DDFS) or to the time of confirmed breast cancer-related death (BCSS) or time of death from any reason (OS). In statistical analysis, *p*-values less than 0.05 were considered significant.

## Results

Our search identified 43 patients fulfilling the latest WHO criteria for NEBC. Out of them, 12 patients were found from Helsinki University Hospital and 31 from Oulu University Hospital. In total 4 patients had distant metastases at the time of the diagnosis, all in bone (Table 1). These primarily metastasized patients were later excluded from the analysis when set against local or locally advanced ductal carcinomas. The mean follow-up time of NEBC was 35.4 months (95% CI 23.5-47.2 months).

**Table 1:** Clinicopathological characteristics of neuroendocrine carcinomas at the time of diagnosis (*n =* 43)

Median age (years)	66.0
Menopausal status	
Premenopausal	2 (4.7%)
Postmenopausal	39 (90.7%)
Not confirmed	2 (4.7%)
T class	
T1	29 (67.4%)
T2	11 (25.6%)
T3	2 (4.7%)
T4	1 (2.3%)
Mean size of primary tumor, mm (95% CI)	25.3 (18.9-31.7)
N class	
N0	24 (55.8%)
N1	10 (23.3%)
N2	5 (11.6%)
N3	2 (4.7%)
Missing	2 (4.7%)
Mean number of lymph node metastases (95% CI)	2.2 (0.4-3.9)
Primary distant metastases	
No	39 (90.7%)
Yes	4 (9.3%)
HER2 expression	
Negative	40 (93.0%)
Positive	2 (4.7%)
Missing	1 (2.3%)
Estrogen receptor expression	
Negative (0%)	1 (2.3%)
Low (1-9%)	0 (0%)
Moderate (10-59%)	0 (0%)
High (>60%)	41 (97.7%)
Missing	1 (2.3%)
Progesterone receptor expression	
Negative (0%)	4 (9.3%)
Low (1-9%)	6 (14.0%)
Moderate (10-59%)	6 (14.0%)
High (>60%)	25 (58.1%)
Missing	2 (4.7%)
Ki-67 expression	
Negative (<5%)	1 (2.3%)
Low (5-14%)	14 (32.6%)
Moderate (15-30%)	15 (34.9%)
High (>30%)	11 (25.6%)
Missing	2 (4.7%)
Multifocal disease	
Yes	15 (35.9%)
No	28 (65.1%)
Synaptophysin expression	
Yes	43 (100%)
No	0 (0%)
Chromogranin expression	
Yes	30 (69.8%)
No	9 (20.9%)
Not available	4 (9.3%)

All (100%) identified patients with neuroendocrine carcinoma had synaptophysin expression >50% of tumor cells, while 30 tumors (69.8%) showed CgA expression. Altogether 30 tumors had a histological diagnosis of DCIS. Both thoracic and abdominal imaging had been performed preoperatively for 28 patients. All patients had either preoperative thoracic and abdominal imaging or a histological diagnosis of DCIS. No evidence of extramammary primary neuroendocrine tumor sites arose during the follow-up.

Clinical and pathological data of the 43 patients with NEBC are described in Table 1. Five patients had a history of earlier breast cancer. From all the NEBC patients, 19 (44.2%) were operated with mastectomy and axillary evacuation, 11 (25.6%) with partial breast resection and sentinel lymph node biopsy and 6 (14.0%) with mastectomy and sentinel biopsy. Six patients (14.0%) were operated with other techniques while one patient was not operated during the follow-up. Thirteen (30.3%) of NEBC patients received adjuvant chemotherapy, 7 having received anthracycline and taxane-based regimen, 3 only anthracycline-based regimen (CEF) and 3 patients were treated with other regimens. Two patients (4.7%) received trastuzumab. Thirty-two (74.4%) had postoperative radiotherapy and 33 (76.7%) adjuvant endocrine therapy, two of them tamoxifen and 31 aromatase inhibitor. Three patients (7.6% of non-metastatic patients) suffered ipsilateral local recurrence during the follow-up, one in axilla, one in mastectomy scar and one in the remaining breast. Distant metastases were not detected at the time of diagnosis. All these patients received adjuvant radiotherapy.

Thirty-nine non-metastatic NEBC were compared to 506 prospectively collected ductal invasive carcinomas with no distant metastases present at the time of diagnosis (Table 2). HER2 was significantly more often negative (94.9%) in NEBC than in ductal carcinomas (86.4%) (*p =* 0.046). All except one NEBC showed strong ER positivity (*p =* 0.0062 compared to ductal carcinomas), one case being triple negative (both ER and PR expression 0%). Ki-67 expression was not significantly different in patients with NEBC or ductal carcinoma (cut-off 15%; *p =* 0.06). The patients with NEBC were older than patients with ductal invasive carcinomas (*p* < 0.0005). All except two patients with NEBC were postmenopausal at the time of diagnosis.

**Table 2:** Neuroendocrine breast carcinomas compared to the prospective set of ductal carcinomas

	NEBC (*n =* 39)	Ductal carcinomas (*n =* 506)	*p*-value
Median age at diagnosis	66.0	57.0	<0.0005
T class			0.40
T1	28 (71.8%)	322 (63.6%)	
T2	9 (23.1%)	167 (33.0%)	
T3	2 (5.1%)	12 (2.4%)	
T4	0 (0.0%)	5 (1.0%)	
N class			0.72
N0	23 (59.0%)	314 (62.1%)	
N1	10 (25.6%)	149 (29.4%)	
N2	5 (12.8%)	41 (8.1%)	
N3	0 (0.0%)	2 (0.4%)	
Missing	1 (2.6%)	0 (0.0%)	
HER2 expression			0.046
Negative	37 (94.9%)	437 (86.4%)	
Positive	1 (2.6%)	69 (13.6%)	
Missing	1 (2.6%)	0 (0.0%)	
Estrogen receptor expression			0.0062
Negative	1 (2.6%)	103 (20.4%)	
Low	0 (0.0%)	18 (3.6%)	
Moderate	0 (0.0%)	25 (4.9%)	
High	37 (94.9%)	360 (71.1%)	
Missing	1 (2.6%)	0 (0.0%)	
Progesterone receptor expression			0.088
Negative	4 (10.3%)	148 (29.2%)	
Low	4 (10.3%)	61 (12.1%)	
Moderate	5 (12.8%)	57 (11.3%)	
High	25 (61.5%)	240 (47.4%)	
Missing	2 (5.1%)	0 (0.0%)	
Ki-67			0.060
Negative	0 (0.0%)	32 (6.3%)	
Low	14 (35.9%)	217 (43.1%)	
Moderate	15 (38.5%)	116 (23.0%)	
High	8 (20.5%)	139 (27.6%)	
Missing	2 (5.1%)	2 (0.4%)	
Adjuvant radiotherapy			0.23
No	8 (20.5%)	66 (13.4%)	
Yes	31 (79.5%)	427 (86.6%)	
Adjuvant chemotherapy			0.00070
No	27 (69.2%)	207 (40.2%)	
Yes	12 (30.8%)	299 (59.1%)	
Adjuvant endocrine therapy			0.015
No	7 (17.9%)	191 (37.7%)	
Yes	32 (82.1%)	315 (62.3%)	

Legend 2. Prognostic factors and adjuvant treatments of neuroendocrine breast carcinomas (NEBC) compared to the prospective set of ductal carcinomas. Primary metastasized cancers have been excluded. Missing values have not been included to the analysis

Fasting plasma CgA levels (median 3.3 nmol/l; range 2.4-9.9 nmol/l) did not correlate to TNM classification, steroid receptor status or HER2 status in NEBC patients. Nevertheless, it correlated with higher age at the time of diagnosis (Spearman’s test *p =* 0.003, correlation coefficient 0.703)

## Survival analyses

**Fig. 1:**
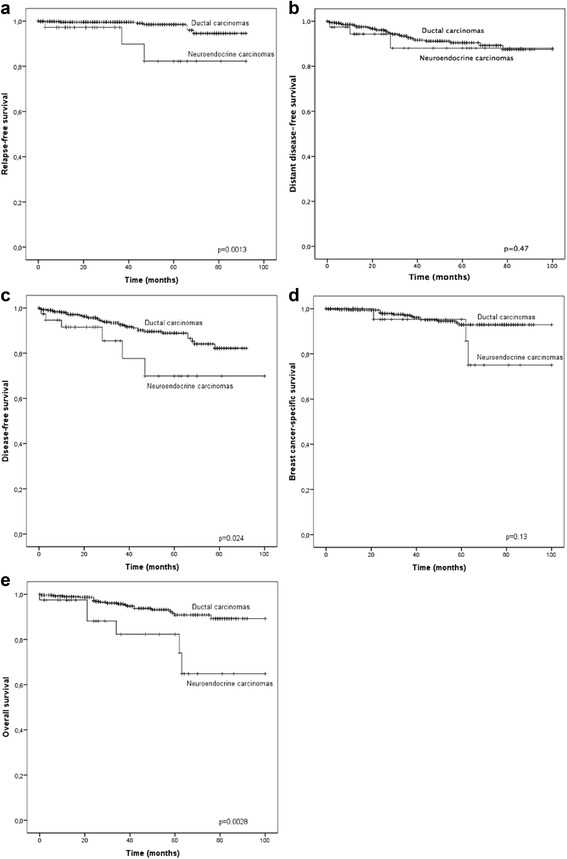
Kaplan-Meier curves comparing neuroendocrine breast carcinomas and ductal breast carcinomas. Legend. Kaplan-Meier curves showing ipsilateral relapse-free survival (**a**), distant disease-free survival (**b**), disease-free survival (**c**), breast cancer-specific survival (**d**) and overall survival (**e**) in non-metastatic neuroendocrine breast carcinomas compared to a prospective set of local or locally advanced ductal breast carcinomas. Crosses indicate censored cases

The prognoses of non-metastatic NEBCs were compared with the prospective cohort of ductal carcinomas (Fig. 1). RFS was significantly worse in patients with NEBC compared to ductal carcinomas (*p =* 0.0013). This difference was reflected in DFS (*p =* 0.024), while no difference was observed in DDFS. BCSS was not significantly different between the groups, but OS was shorter in patients with NEBC (*p =* 0.0028).

Since Ki-67 expression is an essential prognostic factor in ductal breast carcinomas and in gastrointestinal NEC, we assessed its prognostic value in NEBC. Despite being a strong predictor of poorer prognosis in ductal carcinomas (DFS *p =* 0.0006; BCSS *p =* 0.000003), Ki-67 did not have any prognostic significance in NEBC (DFS *p =* 0.904; BCSS *p =* 0.729). Chromogranin expression was not a significant predictor of any survival parameter.

## Discussion

To the best of our knowledge, this is so far the largest study assessing NEBC in the era of the current WHO criteria [1]. Since gastrointestinal NEC is far more prevalent than NEBC, WHO emphasizes the exclusion of extramammary sites before the definite diagnosis of NEBC [1]. Adams et al. [4] recently analysed comprehensively the literature meeting the 2012 WHO criteria for primary neuroendocrine breast tumors. They found 58 published articles, generating data for 108 cases including their own. The mean size of primary tumors in their meta-analysis was 3.7 cm, compared to 2.2 cm of the current study. Lymph node involvement was recorded in 51% and distant metastases in 9% in the papers analysed by Adams et al. in comparison with 39.6% and 9.3% within our material, respectively. TNM data is therefore moderately in line with the previous literature, although our material consisted only cases from 2007, which may contribute to smaller tumor size compared to data consisting also of older cases collected by Adams et al. [4].

Tumor size and lymph node involvement of our NEBC material were also comparable with the prospective set of ductal carcinomas. This is supported by earlier study of Wei et al. [6] in which NEBC had consistent staging compared to a large cohort of invasive breast cancers from Surveillance, Epidemiology and End Results database. Surprisingly, we did not find Ki-67 as a predictor of survival in NEBC, which, however, may be linked to the restricted sample size and limited follow-up of the current material.

The clinical outcome of NEBC has been poorly characterized. Our data, even though having limited sample size, suggest that NEBC patients may suffer from poorer local control despite of adjuvant radiotherapy. Worse RFS did not reflect to BCSS, although NEBC were less often treated with adjuvant chemotherapy. Higher local failure rate cannot be explained by the biological profile of NEBC, which was less aggressive with more ER expression and less HER2 positivity. NEBC patients also received adjuvant radiotherapy as frequently as the patients with ductal carcinomas. In line with the current results, both the 10% local failure rate and high ER positivity/HER2 negativity in NEBC have been previously reported by a retrospective study [6]. Although at the time of the study, WHO diagnostic criteria from 2003 were used [3]. In our material, worse OS of NEBC cases may simply derive from older age of NEBC patient population compared to the patients with ductal carcinoma. The earliest analyses of NEBC suggested dismal prognosis [12, 13] and also more recent retrospective studies have suggested the NEBC patients (diagnosed according to the WHO criteria from 2003) having shorter BCSS and OS compared to the patients at the same stage [6, 14]. From different histological subgroups, small/oat cell carcinoma appears to be an adverse prognostic factor [15]. However, when the most current NEBC classification is used and tumor size and nodal involvement is limited, disease-specific prognosis is rather good [4], which is suggested also by our data. Larger material or meta-analysis would still be required to more definitely conclude the impact of NEBC differentiation to prognosis.

The reported or estimated incidence for NEBC has been <1% of all breast cancers [1, 2]. There were on average 273 breast cancer cases per year in the hospital district of Oulu University Hospital in 2009–2013 [16]. On average, 31 NEBC cases in 8.5 years in Oulu totals 3.6 cases per year. This is 1.3% of all breast cancers being somewhat higher compared to previously reported incidences. On the other hand, in Helsinki University Hospital area there were on average 1367 new breast cancer cases per year. Therefore corresponding incidence for NEBC was 0.1% of all breast cancers, 13-fold difference compared to the incidence in Oulu University Hospital area. This is most likely due to variation of neuroendocrine marker immunostaining prevalence between hospitals. Indeed, when in some studies neuroendocrine markers have been screened from unselected breast carcinomas, up to 10.4% of all breast cancers showed some synaptophysin or chromogranin A expression [17].

## Conclusions

In summary, there seems to be a huge variation in the incidence of NEBC within Finland, which probably cannot be explained by true geographical variation. There is a severe need for harmonization of NEBC diagnostics in order to assess its pathobiological and clinical features in more detail in larger and optimally in prospective studies. The use of chromogranin and synaptophysin immunohistochemistry in a case of even lower suspicion of NEBC could help to find these cancers more precisely. Still the majority of neuroendocrine cancers have origin outside breast and the exclusion on intermammary sites is of the utmost importance. Although the best surgical and oncological management of NEBC still remains unknown, future studies should pay attention to possible higher tendency of high local failure rates.

## Abbreviation

BCSS: Breast cancer-specific survival
CEF: Cyclophosphamide-epirubicin-5-fluorouracil
CgA: Chromogranin A
DCIS: Ductal in situ carcinoma
DDFS: Distant disease-free survival
DFS: Disease-free survival
ER: Estrogen receptor
NE markers: Neuroendocrine markers
NEBC: Breast carcinoma with neuroendocrine features
NEC: Carcinoma with neuroendocrine features
OS: Overall survival
PR: Progesterone receptor
RFS: Relapse-free survival
WHO: World Health Organization

## References

[1] Lakhani SR, Ellis I, Schnitt S (2012). WHO classification of tumors of the breast. International agency for research on cancer.

[2] López-Bonet E, Alonso-Ruano M, Barraza G, Vazquez-Martin A, Bernadó L, Menendez JA (2008). Solid neuroendocrine breast carcinomas: incidence, clinico-pathological features and immunohistochemical profiling. Oncol Rep..

[3] Tavassoli FA, Devilee P (2003). World Health Organization classification of tumors, pathology and genetics of tumors of the breast and female genital organs.

[4] Adams RW, Dyson P, Barthelmes L (2014). Neuroendocrine breast tumors: breast cancer or neuroendocrine cancer presenting in the breast?. Breast.

[5] Miremadi A, Pinder SE, Lee AH, Bell JA, Paish EC, Wencyk P (2002). Neuroendocrine differentiation and prognosis in breast adenocarcinoma. Histopathology.

[6] Wei B, Ding T, Xing Y, Wei W, Tian Z, Tang F (2010). Invasive neuroendocrine carcinoma of the breast: A distinctive subtype of aggressive mammary carcinoma. Cancer.

[7] Wachter DL, Hartmann A, Beckmann MW, Fasching PA, Hein A, Bayer CM (2014). Expression of neuroendocrine markers in different molecular subtypes of breast carcinoma. Biomed Res Int..

[8] Ang D, Ballard M, Beadling C, Warrick A, Schilling A, West RB (2015). Novel mutations in neuroendocrine carcinoma of the breast: possible therapeutic targets. Appl Immunohistochem Mol Morphol.

[9] Weigelt B, Geyer FC, Horlings HM, Kreike B, Halfwerk H, Reis-Filho JS (2009). Mucinous and neuroendocrine breast carcinomas are transcriptionally distinct from invasive ductal carcinomas of no special type. Mod Pathol.

[10] Karihtala P, Mäntyniemi A, Kang SW, Kinnula VL, Soini Y (2003). Peroxiredoxins in breast carcinoma. Clin Cancer Res.

[11] Isola J, Tanner M, Forsyth A, Cooke TG, Watters AD, Bartlett JMS (2004). Interlaboratory comparison of HER-2 oncogene amplification as detected by chromogenic and fluorescence in situ hybridization. Clin Cancer Res.

[12] Wade PM, Mills SE, Read M, Cloud W, Lambert MJ, Smith RE (1983). Small cell neuroendocrine (oat cell) carcinoma of the breast. Cancer.

[13] Papotti M, Macrì L, Finzi G, Capella C, Eusebi V, Bussolati G (1989). Neuroendocrine differentiation in carcinomas of the breast: a study of 51 cases. Semin Diagn Pathol.

[14] Wang J, Wei B, Albarracin CT, Hu J, Abraham SC, Wu Y (2014). Invasive neuroendocrine carcinoma of the breast: a population-based study from the surveillance, epidemiology and end results (SEER) database. BMC Cancer.

[15] Cloyd JM, Yang RL, Allison KH, Norton JA, Hernandez-Boussard T, Wapnir IL (2014). Impact of histological subtype on long-term outcomes of neuroendocrine carcinoma of the breast. Breast Cancer Res Treat.

[16] Finnish Cancer Registry, Cancer Statistics at www.cancerregistry.fi. Accessed 8 Oct 2015.

[17] Bogina G, Munari E, Brunelli M, Bortesi L, Marconi M, Sommaggio M (2015). Neuroendocrine differentiation in breast carcinoma: clinicopathological features and outcome. Histopathology.

